# Loneliness as a risk factor for frailty transition among older Chinese people

**DOI:** 10.1186/s12877-020-01714-5

**Published:** 2020-08-24

**Authors:** Sha Sha, Yuebin Xu, Lin Chen

**Affiliations:** 1 School of Social Development and Public Policy, Beijing Normal University, Beijing, 100875 China; 2 Institute of advanced Studies in Humanities and Social Sciences, Beijing Normal University at Zhuhai, Zhuhai, 519087 China; 3 International Business Faculty, Beijing Normal University, Zhuhai, 519087 China

**Keywords:** Loneliness, Frailty, Frailty transition, Longitudinal, Older people, Gender difference

## Abstract

**Background:**

Previous literature has reported that loneliness is a strong predictor of frailty risk. However, less is known about the role of loneliness in frailty transition types. This study aimed to examine whether and how loneliness are related to frailty transition among older Chinese people.

**Methods:**

Our study used participants (aged ≥60 years) from 2008/2009, 2011/2012 and 2014 waves of the Chinese Longitudinal Healthy Longevity Survey (CLHLS). Loneliness was assessed by a single question asking how often the respondent feels lonely. The FRAIL Scale was created to measure physical frailty for our study, and frailty was also assessed by a broader definition of the frailty index. Frailty transition as an outcome variable has been designed as two types according to the measurement of frailty.

**Results:**

Greater loneliness at baseline reduced the possibility of remaining in a robust or prefrail physical frailty state after 3 years (OR = 0.78, 95%CI: 0.68–0.91, *p* < 0.01). Greater loneliness was associated with an increased risk of worsening physical frailty over time: compared with those who had never felt lonely, the odds ratios for people who often felt lonely were 1.19 (95%CI: 1.01–1.41, *p* < 0.05) after 3 years and 1.34 (95%CI: 1.08–1.66, *p* < 0.01) after 6 years. The association between loneliness and change in the frailty index differed in the survey periods: loneliness at baseline was found to increase the possibility of participants remaining in frailty (seldom loneliness: OR = 1.78, 95%CI: 1.25–2.55, *p* < 0.01; often loneliness: OR = 1.74, 95%CI: 1.21–2.50, p < 0.01) after 6 years, but no significance was shown in the 3-year follow up. Additionally, loneliness at baselines was significantly associated with frailty transition at follow up among the male participants. However, a similar association was not observed among the female participants.

**Conclusion:**

Older people with a high level of loneliness tend to be frail in the future, and greater loneliness is related to an increased risk of worsening frailty and remaining frail. Male elderly with a high level of loneliness were more likely to have a worse frailty transition than female elderly in China.

## Background

Populations worldwide are rapidly aging, which presents a particularly severe challenge in China, a country where the population is aging at a significantly faster rate than other low- and middle-income countries [1]. According to data published by the National Bureau of Statistics of China, 17.3% of the total population were aged 60 years and older in 2017. However, due to the implementation of the one-child policy in the earlier decades and recent increasing population mobility, the family size in China has been declining substantially, leading to over 50% of the urban and 60% of rural elderly living in empty-nest households, and the proportion of older people living alone in China was 12.5% in 2010, an increase by 30% in the prior two decades [2, 3]. These findings all have important implications for the health and social care of the elderly.

Frailty is the most outstanding expression of population aging [4]. It is a syndrome that predicts vulnerability to adverse outcomes and is recognized as a dynamic state with the potential for reversibility [4–7]. There are currently two major models of frailty: first, the frailty phenotype model that views frailty from the physiological systems and defines frailty as several biological syndromes [8]; second, the frailty deficit model measures frailty as problems resulting from a multidimensional system, including biological, physiological and psychological [9]. Despite a sizeable literature on the adverse outcomes of frailty, such as falls [10], disability [11, 12], hospitalization [13], institutional care [14, 15], and mortality [16–18], relatively little is known about the transition of frailty in older people. Although frailty is inevitable with increasing age [19], it is not irreversible but agreeable to be a dynamic process involving improvement and natural procession [4].

Loneliness is a common and dissatisfaction feeling of one’s social relationship that is presently becoming a serious public health issue for older people [20, 21]. Loneliness has been observed to be associated with subsequent adverse outcomes, such as mortality [22, 23], comorbidity [24, 25], poor functional ability [26, 27], depression [28], and cognitive decline [29]. At the biological level, many studies have found that the feeling of loneliness is associated with increased blood pressure [30, 31], increased risk of cardio-cerebrovascular and inflammatory diseases [32–34], impaired immune function [35], and increased likelihood of sarcopenia [36].

Frailty, defined by the phenotype model or frailty index, is associated with loneliness [37–39]. A cross-sectional study of Mexican community-dwelling elderly found that loneliness was independently associated with frailty [37]. The cohort study discovered that the relationship between frailty and loneliness might be bidirectional: loneliness was related to the change in frailty status, and vice versa [38, 40]. However, studies on the association between loneliness and frailty were focused on loneliness and frailty risk, and none have specified the association between loneliness and frailty transition types, including remaining frail, worsening or improving in frailty status. Furthermore, the existing research was based mostly on western society; much less is known for older people in other studies.

A study of Chinese older adults had reported that 51.2 and 7.0% of older adults aged 60 years and older were prefrail and frail, respectively [41]. However, little research exists on frailty transitions in China. It was reported that about 30.4% of participants had transitioned between different frailty statuses in 2002–2005 [42]. Additionally, one study showed that loneliness was related to culture and social policies [43]. People from collectivist cultures are more likely to feel lonely [44]. It was reported that about 30% of Chinese older adults reported feelings of loneliness [45]. We speculated that loneliness could have more influence on frailty transition in China. Gender difference in frailty and loneliness is well known [46, 47]. Women tend to have a higher incidence of frailty than men [48, 49], and it was suggested that this might be attributable to both biological and socioeconomic factors [50]. Studies have shown that loneliness is strongly associated with adverse health conditions in men and women [51, 52]. Thus, it can be assumed that the association between loneliness and frailty transitions is gender related.

Our study aimed to examine the association between loneliness and frailty transition among older adults older than 60 years in China. We generated two hypotheses: 1) loneliness is related to frailty transition, and 2) the relationship between loneliness and frailty is different by gender. We believe that our study would help to close the gap in the existing literature by using a nationally representative longitudinal sample in China, and the results would also be useful in informing policy making in health and social care.

## Method

### Data

The data came from the Chinese Longitudinal Healthy Longevity Survey (CLHLS), which is the first and largest nationwide longitudinal survey in China. The survey is designed to investigate the determinants of the health and longevity of older adults in China. Thus far, information has been collected in half of the randomly selected cities/counties in 23 of 31 provinces in China, with a total of 113,000 households being interviewed. The CLHLS was initiated in 1998, and follow-up interviews were conducted in 2000, 2002, 2005, 2008/2009, 2011/2012, 2014 and 2017/2018 [53]. The questionnaire contained information about demographics, lifestyle, diet, self-reported health, psychological health, activities of daily living (ADL) and instrumental activities of daily living (IADL). The rationales, more details of the survey design, and data quality were published elsewhere [54].

**Fig. 1:**
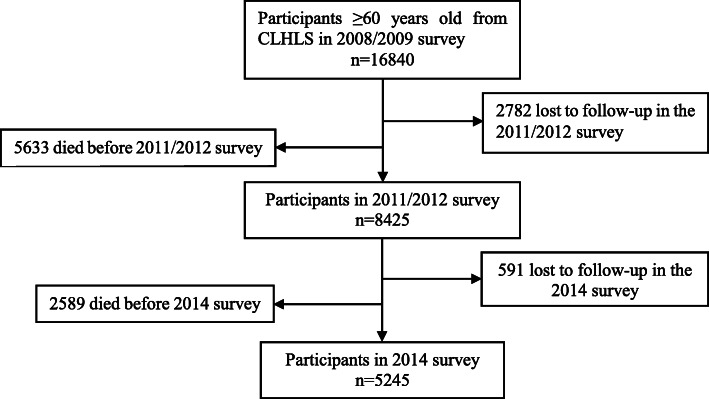
Flow diagram of participants

Our study utilized the participants of CLHLS in 2008/2009, 2011/2012 and 2014 to conduct two cohorts. Briefly, among the 16,840 participants (aged ≥60 years) in 2008/2009, 2782 (17%) were lost to follow up and 5633 (33%) died before the 2011/2012 survey. We excluded those lost to follow-up due to their unknown information and removed those who died before the follow-up to eliminate the effect of mortality. Finally, a valid sample size of 8425 participants was analyzed for the 3-year follow-up period (2008–2011). Among the 16,840 participants (aged ≥60) in 2008/2009, 8415 (50%) died or were lost to follow up in the 2008–2011 waves, 591 (4%) were lost to follow up in the 2014 survey and 2589 (15%) died before the 2014 survey. In total, 5245 (31%) participants were alive for analysis for the 6-year follow-up period (2008–2014). The flowchart of the study is shown in Fig. 1.

### Measures

#### Loneliness

Loneliness was measured with a single question asking how often the respondent feels lonely. The 5-point response scale ranged from “never” to “always”. Single-item questions are sometimes known as self-rating measures of loneliness because they can ask directly for the individual’s assessment of how lonely they feel. The single question of loneliness has been used widely [55–57] and has proven to be valid and highly correlated with multi-item loneliness scales [58, 59]. Because the question on loneliness in CLHLS is highly skewed with fewer respondents in the “always” and “often” categories, we classified “sometimes”, “often” and “always” into one category and “seldom” and “never” into another category to show the level of loneliness.

#### Frailty status

The FRAIL Scale [60] was created to measure physical frailty for our study. It comprises 5 simple questions to assess the presence of fatigue, muscle resistance, aerobic capacity, disease burden, and weight loss [61]. Those who met three or more components were defined as frail, those with 1 or 2 components were deemed as prefrail, and those without any were defined as robust [60, 62]. Based on the CLHLS questionnaire design, we made some adjustments to the FRAIL Scale indicators. Each item in the FRAIL Scale was dichotomized and mapped to the interval 0–1. Fatigue was measured using the question of “Do you feel the older you get, the more useless you are?” The analysis codes “never”, “seldom”, and “sometimes” as 0 and 1 otherwise. Resistance was measured as “Can you continuously crouch and stand up three times?” and ambulation as “Can you walk continuously for 1 kilometer at a time by yourself?”. For the two variables, the analysis recodes 0 for “without assistance” and 1 otherwise. Illness was measured by self-reporting more than 5 types of illness and was coded as 1. Loss of weight was measured by BMI (weight (in kilograms)/height (in meters) ^2^) using the same cutoff points of underweight (< 18.5).

Our study also used another model of the frailty index to measure the dimensions of frailty, in which at least 30 deficits are needed [63]. We used 37 indicators of various dimensions of the frailty status, coded as 1 when the deficits occurred and 2 if the respondents had a serious illness that caused him/her to be hospitalized or bedridden two or more times [18, 64, 65]. A high value in the frailty index indicated poorer frailty. We also classified the continuous frailty index into nonfrail (FI ≤0.21) and frail (FI > 0.21) based on previous studies [42, 66]. A full description of the frailty index can be found in Table 1.

**Table 1:** List of items included in a frailty index

NO.	Items
1	ADLs: needs assistant in bathing
2	ADLs: needs assistant in dressing
3	ADLs: needs assistant in toileting
4	ADLs: needs assistant in indoor transferring
5	ADLs: needs assistant in continence
6	ADLs: needs assistant in eating
7	IADLs: unable to visit neighbors by himself/herself
8	IADLs: unable to go shopping by himself/herself
9	IADLs: unable to cook a meal by himself/herself
10	IADLs: unable to wash clothing by himself/herself
11	IADLs: unable to walk continuously for 1 km at a time by himself/herself
12	IADLs: unable to lift a weight of 5 kg
13	IADLs: unable to continuously crouch and stand up three times
14	IADLs: unable to take public transportation by himself/herself
15	Cognitive impairment (based on Mini Mental State Examination)
16	Poor self-reported health
17	Health state compared to past year
18	Poor interviewer-rated health
19	Vision loss
20	Psychological distress (based on usefulness, fearfulness)
21	Number of serious illnesses in the past 2 years^a^
22	Suffering from hypertension
23	Suffering from diabetes
24	Suffering from heart disease
25	Suffering from stroke or cerebrovascular disease
26	Suffering from bronchitis, emphysema, pneumonia, asthma
27	Suffering from tuberculosis
28	Suffering from cataract
29	Suffering from cancer
30	Suffering from Parkinson’s disease
31	Suffering from arthritis
32	Suffering from dementia
33	Functional limitations: unable to put hand behind neck
34	Functional limitations: unable to put hand behind lower neck
35	Functional limitations: unable to raise arm upright
36	Functional limitations: unable to stand up from sitting in a chair
37	Functional limitations: unable to pick up a book from floor

^a^Two or more serious illnesses in the past 2 years are assigned a value of 2

#### Frailty transitions

The change in frailty status between 2008 and the follow up in 2011 and 2014 was used as the outcome. Frailty transitions were created as two types in our study because we used two frailty models.

Four transitions between the physical frailty states were designed in our study according to the Frail Scale: remaining robust or prefrail, indicating that the elderly have remained healthy to some extent; improvement, indicating improvement or a change from prefrail to robust or from frail to robust or prefrail; worsening, indicating a transition to greater frailty; and remaining frail, indicating that the elderly have remained unhealthy.

The change types in the frailty index were classified into four categories: remaining nonfrail, indicating that the frailty index of the elderly was under 0.21 during the period; worsening, indicating that the frailty index of the elderly had changed from nonfrail to frail; improvement, indicating that the frailty scores declined to nonfrail in the follow-up year; remaining frail, indicating that the frailty index remained frail in follow-up.

#### Covariates

Covariates were measured at baseline and included age, gender, living arrangement, residential area, education, relative economic status, smoking, drinking alcohol and the baseline physical frailty state.

Living arrangement was coded as 0 if participants were living independently and 1 otherwise. The residential area was commonly used in studies about China because urban and rural areas differ greatly in socioeconomic development [67]. The participants were asked about their years of education, which is used as a continuous variable in our study. The relative economic status was measured with the question: “How do you rate your economic status compared with others in your local area?”. The response was classified into three categories and we reverse-coded them so that higher categories indicated higher economic status (1 = poor; 2 = so so; 3 = rich). The frailty transition between frailty states was highly dependent on the preceding frailty state [5], and the baseline frailty states were viewed as the number of components of the frail scale present in the baseline.

### Analytical sample

In the 3-year period, 8425 participants were included in the 2008–2011 waves. The analysis of loneliness with frailty transition was based on 5746 (68%) re-interviewed participants with completed data. The analysis of frailty index change was based on 5618 (67%) re-interviewed participants with completed data.

In the 6-year period, 5245 participants were included in the 2008–2014 waves. The analysis of loneliness with frailty transition was based on 3548 (68%) participants with completed data by re-interviewed participants. The analysis of the frailty index change was based on 3288 (63%) participants with completed data among re-interviewed participants.

### Method of analysis

Descriptive statistics at baseline were summarized using means (±standard deviation) or counts (percentages). Logistic regression was used to derive the odds ratios of loneliness for the physical frailty transition types and frailty index change types. Logistic regression was also conducted for women and men separately to explore gender differences in the relationships between frailty transition and loneliness. Estimates are shown, adjusted for age and the number of components of baseline physical frailty and others. All the analyses were performed using the statistical package STATA version 15.0. A *p*-value< 0.05 was deemed statistically significant.

## Results

### Descriptive characteristics

Table 2 summarizes the participant characteristics at baseline during the survey period. In the 2008–2011 waves, the prevalence of often loneliness at baseline was 28.2%, which slightly decreased to 25.8% in the 2008–2014 waves. Compared with the 6-year follow-up, participants in the 3-year period were older, more likely to be female, had less education, were more likely to live independently, smoked and drank less, and were frailer both in the physical frailty scale and frailty index at baseline.

**Table 2:** Characteristic of the participants at baseline in two survey periods

	3-year period (2008–2011)	6-year period(2008–2014)
	(*N* = 8425)	(*N* = 5245)
Age, mean (SD)	82.6 (11.0)	79.1 (10.1)
Gender:female,n(%)	4607 (54.7)	2800 (53.4)
Education year, mean (SD)	2.4 (3.6)	2.6 (3.6)
Residenc:Rural, n(%)	5207 (61.8)	3319 (63.3)
Living arrangement: independently, n(%)	1368 (16.2)	843 (16.1)
Relative economic status, n(%)		
rich	1467 (17.4)	873 (16.7)
so so	5778 (68.7)	3622 (69.2)
poor	1162 (13.8)	740 (14.1)
Current somker, n(%)	1710 (20.3)	1178 (22.5)
Current drinker, n(%)	1670 (19.8)	1114 (21.2)
Loneliness, n(%)		
never	3262 (41.9)	2208 (43.8)
seldom	2330 (29.9)	1532 (30.4)
often	2195 (28.2)	1301 (25.8)
No. of components of frail scale at baseline, mean (SD)	1.20 (1.2)	0.98 (1.1)
Frailty index score at baseline, mean (SD)	0.13 (0.1)	0.11 (0.1)

### Frailty transitions

Table 3 shows the transition in frailty status between the baseline and follow-up. In the 2008 and 2011 waves, nearly half (49.3%) of the participants transitioned between different frailty states (robust, prefrail and frail), 2605 (45.3%) remained robust or prefrail, and 5.3% remained frail at the follow up. Of the total participants in the 2008 and 2014 waves, more than half (51.0%) changed, 1617 (45.6%) had maintained a robust or prefrail state, and 3.5% remained in a frail state in the follow-up visit. Overall, the frailty transition was similar between the two periods.

**Table 3:** Physical frailty transitions between baseline and follow-up, n (%)

	3 -year period (2008–2011)			6-year period (2008–2014)		
	Total	female	male	Total	female	male
Remaining robust and prefrail	2605 (45.3)	1160 (40.0)	1445 (50.8)	1617 (45.6)	731 (40.9)	886 (50.3)
Worsening	1649 (28.7)	870 (30.0)	779 (27.4)	1132 (31.9)	586 (32.8)	546 (31.0)
Improvement	1185 (20.6)	630 (21.7)	555 (19.5)	676 (19.1)	371 (20.8)	305 (17.3)
Remaining frail	307 (5.3)	241 (8.3)	66 (2.3)	123 (3.5)	98 (5.5)	25 (1.4)
Total	5746	2901	2845	3548	1786	1762

Notes: chi-squared test for physical frailty transitions by gender in 3-year period: *p* < 0.0001

chi-squared test for physical frailty transitions by gender in 6-year period: *p* < 0.0001

There was a clear difference between the distribution of frailty transition among female and male participants. In the two periods, nearly half of the male participants remained in the robust or prefrail state, whereas about 40% of the female participants remained at the same level. More female participants worsened in the physical frailty state than male participants, and male participants had a lower prevalence of remaining frail than female participants. However, the female participants showed a higher probability of recovering from greater frailty than the male participants. More than a fifth of the female participants had improved from a greater frailty state in both periods, while 17.3 and 19.5% of male participants had improved in the two periods, respectively.

### Physical frailty transition as an outcome

The associations between physical frailty transition and loneliness are shown in Table 4. In the remaining robust or prefrail group, after adjusting for age, gender, number of components in the frail scale and others at baseline, significant trends in the remaining frail state were associated with a high level of loneliness observed in the 3-year period: compared with the never lonely participants, those who often felt lonely were unlikely to remain in the robust or prefrail state (OR = 0.78, 95%CI: 0.68–0.91, *p* < 0.01). In the worsening group, loneliness was a significant risk factor in that a high level of loneliness was associated with worsened frailty states over time years (3-year period: OR = 1.19, 95%CI: 1.01–1.41, *p* < 0.05; 6-year period: OR = 1.34, 95%CI: 1.08–1.66, *p* < 0.01). In the improvement group, loneliness showed no significant influence on frailty transition. Loneliness at baseline was positively associated with remaining frail in the 6-year period (seldom loneliness: OR = 2.47, 95%CI: 1.25–4.85, *p* < 0.01) but no significant association was shown in the 3-year period.

**Table 4:** Odds ratios (95% CI) for physical frailty transitions and loneliness

	3 -year period (2008–2011)				6-year period (2008–2014)			
	Remaining robust and prefrail	Worsening	Improvement	Remaining frail	Remaining robust and prefrail	Worsening	Improvement	Remaining frail
Total								
never	Ref.	Ref.	Ref.	Ref.	Ref.	Ref.	Ref.	Ref.
seldom	0.99	1.05	1.03	1.15	0.98	1.02	0.99	2.47**
	(0.87–1.13)	(0.90–1.21)	(0.86–1.23)	(0.76–1.73)	(0.83–1.15)	(0.85–1.22)	(0.77–1.26)	(1.25–4.85)
often	0.78**	1.19*	1.14	1.00	0.84	1.34**	0.85	1.88
	(0.68–0.91)	(1.01–1.41)	(0.94–1.39)	(0.67–1.51)	(0.70–1.01)	(1.08–1.66)	(0.65–1.12)	(0.93–3.79)
Female								
never	Ref.	Ref.	Ref.	Ref.	Ref.	Ref.	Ref.	Ref.
seldom	1.00	1.01	1.02	1.17	1.01	1.12	0.77	1.77
	(0.83–1.21)	(0.82–1.25)	(0.79–1.32)	(0.73–1.87)	(0.80–1.28)	(0.86–1.46)	(0.55–1.08)	(0.83–3.77)
often	0.85	1.04	1.07	0.96	0.94	1.20	0.78	1.40
	(0.69–1.05)	(0.82–1.32)	(0.82–1.40)	(0.60–1.53)	(0.73–1.21)	(0.89–1.60)	(0.55–1.10)	(0.65–3.02)
Male								
never	Ref.	Ref.	Ref.	Ref.	Ref.	Ref.	Ref.	Ref.
seldom	0.99	1.08	1.03	1.35	0.96	0.92	1.28	12.68*
	(0.83–1.18)	(0.88–1.33)	(0.80–1.34)	(0.55–3.32)	(0.77–1.20)	(0.71–1.18)	(0.90–1.82)	(1.66–96.71)
often	0.73**	1.37*	1.25	1.37	0.75*	1.54**	0.94	8.89
	(0.59–0.89)	(1.07–1.75)	(0.93–1.68)	(0.58–3.24)	(0.57–0.99)	(1.13–2.11)	(0.61–1.44)	(0.75–105.30)

***P < 0.001, **P < 0.01, *P < 0.05;

Sample size: 2008–2011 waves: total participants: 5689; for female participants:2866; for male participants:2823;

2011–2014 waves: total participants: 3529; for female participants:1776; for male participants:1753

Notes: Model had been adjusted for age, components number in the frail scale at baseline, residence, education year, living arrangement, relative economic status, smoking and drinking alcohol at baseline. In total participants, adjustment for gender was also performed

We also investigated whether the association between loneliness and physical frailty transition differed by gender. Male participants who felt lonely often were negatively related to remaining robust and prefrail (3-year period: OR = 0.73, 95%CI: 0.59–0.89, *p* < 0.01; 6-year period: OR = 0.75, 95%CI: 0.57–0.99, *p* < 0.05) and were positively related to worsening frailty (3-year period: OR = 1.37, 95%CI: 1.07–1.75, *p* < 0.05; 6-year period: OR = 1.54, 95%CI: 1.13–2.11, *p* < 0.01) in the two survey periods. Loneliness of male participants was also found to be related to remaining frail after 6 years (seldom loneliness: OR = 12.68; 95%CI: 1.66–96.71, *p* < 0.05).

### Frailty index as an outcome

Table 5 presents the odds ratios (95%CI) for the change in the frailty index and loneliness. Loneliness at baseline was observed to be a protective factor for the improvement in the frailty index only in the 3-year period (seldom loneliness: OR = 1.42, 95%CI: 1.04–1.95, *p* < 0.05; often loneliness: OR = 1.50, 95%CI: 1.08–2.08, *p* < 0.05). Regarding the 6-year period, loneliness at baselines was found to increase the possibility of participants to remain frail (seldom loneliness: OR = 1.78, 95%CI: 1.25–2.55, *p* < 0.01; often loneliness: OR = 1.74, 95%CI: 1.21–2.50, *p* < 0.01) after 6 years.

**Table 5:** Odds ratios (95% CI) for transition type in frailty index and loneliness

	3 -year period (2008–2011)				6-year period (2008–2014)			
	Remaining nonfrail	Worsening	Improvement	Remaining frail	Remaining nonfrail	Worsening	Improvement	Remaining frail
Total								
never	Ref.	Ref.	Ref.	Ref.	Ref.	Ref.	Ref.	Ref.
seldom	1.03	0.94	1.42*	1.03	0.99	0.93	0.76	1.78**
	(0.87–1.20)	(0.79–1.11)	(1.04–1.95)	(0.82–1.30)	(0.82–1.20)	(0.77–1.13)	(0.47–1.22)	(1.25–2.55)
often	0.89	0.96	1.50*	1.07	0.83	1.00	0.88	1.74**
	(0.75–1.05)	(0.80–1.16)	(1.08–2.08)	(0.84–1.36)	(0.67–1.02)	(0.80–1.24)	(0.54–1.42)	(1.21–2.50)
Female								
never	Ref.	Ref.	Ref.	Ref.	Ref.	Ref.	Ref.	Ref.
seldom	1.20	0.89	1.12	0.93	1.02	0.98	0.65	1.39
	(0.96–1.49)	(0.71–1.11)	(0.73–1.72)	(0.70–1.23)	(0.78–1.33)	(0.76–1.28)	(0.35–1.22)	(0.92–2.10)
often	0.96	0.87	1.54*	0.98	0.77	1.04	0.93	1.50
	(0.77–1.21)	(0.68–1.10)	(1.02–2.31)	(0.73–1.31)	(0.58–1.01)	(0.79–1.38)	(0.51–1.67)	(0.99–2.27)
Male								
never	Ref.	Ref.	Ref.	Ref.	Ref.	Ref.	Ref.	Ref.
seldom	0.87	0.97	2.00**	1.30	0.96	0.88	0.95	3.58***
	(0.69–1.10)	(0.76–1.25)	(1.23–3.24)	(0.86–1.96)	(0.73–1.27)	(0.66–1.16)	(0.46–1.98)	(1.73–7.41)
often	0.84	1.05	1.41	1.35	0.94	0.95	0.77	2.70**
	(0.64–1.09)	(0.79–1.39)	(0.81–2.44)	(0.88–2.05)	(0.68–1.30)	(0.68–1.33)	(0.34–1.77)	(1.27–5.76)

***P < 0.001, **P < 0.01, *P < 0.05

Sample size: 2008–2011 waves: total participants: 5548; female participants:2833; male participants:2715;

2011–2014 waves: total participants: 3381; for female participants:1737; for male participants:1644

Notes: Model had been adjusted for age, components number of frail scale at baseline, residence, education year, living arrangement, relative economic status, smoking and drinking alcohol at baseline. In total participants, adjustment for gender was also performed

Gender differences were also found in the transition type of frailty index and loneliness. Loneliness in male participants was related to remaining frail in the 6-year period (seldom loneliness: OR = 3.58, 95%CI: 1.73–7.41, *p* < 0.001; often loneliness: OR = 2.70, 95%CI: 1.27–5.76, *p* < 0.01), but no significant relationship was found in female participants. A high level of loneliness in female participants was associated with improvement in frailty in the 3-year period (OR = 1.54; 95%CI: 1.02–2.31; *p* < 0.05). The association between loneliness and the frailty index in male participants was the same ash that found in all participants, except that no relationship was observed with the improvement in the frailty index in the 3-year period in male participants with seldom loneliness.

## Discussion

The present study investigated the association between loneliness and frailty transitions. We used the 2008/2009, 2011/2012 and 2014 surveys of CLHLS for the analysis, focusing on the difference in the relationships between the male and female participants.

Nearly half of the participants remained in the robust or prefrail state in the follow-up years, regardless of gender. The percentage was higher than that in a previous study in China reporting that 39.6% of participants remained in the robust or prefrail state in 2002–2005 [42], while the percentage was close to the pooled frailty transition rates among 16 cohorts from 2010 to 2018 [68]. This difference may be due to the baseline time, variations in the follow-up year and measurement of frailty. Obviously, they may indicate that a window exists during which early interventions may be taken for the elderly to maintain their health status as much as possible. Furthermore, we found that changes from worsening to greater physical frailty tended to be more common than recovering from greater physical frailty, and this pattern of transition was consistent with that in a previous study [42, 68]. Evidence on gender differences in frailty transition is rare. One study of older people in Hongkong between 2001 and 2003 found that women were less likely to decline in frailty status than men [69], whereas a longitudinal study in San Antonio did not find men to be at higher risk of declining frailty status [70]. Another cohort study in older Italian adults found that women were more likely to progress into worsened physical frailty than men with a mean follow-up of 4.4 years [71]. Our study showed that, compared with men, women were more likely to change frailty status, either improving or worsening, a finding that agrees with a recent systematic review [68] and requires further confirmation by more studies. Thus, frailty interventions may have different efficacies in men and women.

Our study designed four physical frailty transition types—remaining robust or prefrail, improvement, worsening and remaining frail—to ascertain the specific relationship between loneliness and frailty transition. Previous studies have identified loneliness to be related to frailty [37, 38]. In our study, we found that greater loneliness reduced the possibility of remaining robust or prefrail physical frailty after 3 years, a finding that is consistent with a study in England, in which greater loneliness was found to be associated with increased risk of physical frailty around 4 years later [40]. Additionally, our study used two follow-up periods to validate the relationship. We found that loneliness increased the risk of older people with worsening frailty as well as those remaining frail after 6 years. However but no significant relationship was shown in the 3-year period. These findings may indicate that loneliness not only increases the possibility of frail in older adults but also increases the likelihood of older adults becoming frailer and chronically frail.

Our study also used another model of frailty, the frailty index, to further verify the results on the relationship between loneliness and frailty transition. We found a clear difference in the association between the levels of loneliness and frailty index between the two survey periods. In the 3-year follow-up, we found that the loneliness at baseline was related to recovering the frailty status in the frailty index. This may be explained by the frailty status at baseline because a severe baseline frailty status is more likely to be improved during the follow-up period. Correspondingly, the relationship between frailty improvement and loneliness was no longer significant in the 6-year follow-up, suggesting that shorter follow-up periods provide more time for older people to change their frailty status. During the 6-year follow-up, loneliness was found to be positively related to remaining in the frail status, but no significant relationship was found in the 3-year follow-up. This finding was supported by a previous study showing that a longer follow-up period was associated with lower rates of remaining in the same frailty state [68]. Additionally, the relationship between frailty index transition and loneliness differed from that between loneliness and physical frailty. For example, loneliness was found not to be associated with worsening in the frailty index, but with worsening physical frailty over time. The broader definition of the frailty index may not have the same risk factors as those of physical frailty [40]. Our findings confirmed this point, and more studies are needed to consolidate the possibility.

Our study also showed that loneliness varied by gender [52]. Low resilience was associated significantly with loneliness and was more pronounced in males [51]. Conversely, high resilience can be a protective factor in facilitating older people to maintain their health status [72, 73]. Previous research also indicated the stressful impact of loneliness on men, as manifested by increased inflammatory responses [74]. The inflammatory response has a specific physiological basis in the geriatric syndrome of frailty [75], which may be a mechanism underlying gender differences in the relationship between loneliness and physical frailty. Moreover, women tended to have more informal networks, which may lead to more social support, whereas men maintain their social relationships more from the public sphere, which may not always be socially supportive [59, 76]. Our study indicated that the male participants were more sensitive to the relationship between loneliness and frailty transitions, defined by either physical frailty or the frailty index. Most of the significant relationships observed in the male participants were identical to the findings for all participants. Thus, loneliness in males warrants more attention.

Given that frailty in older adults may be modifiable, our findings have potential implications for both health and social care policy and practice. First, our study provides a picture of the frailty transition among older people in China, highlighting the importance of early interventions for older people to maintain or improve their health statuses, particularly for those in the robust or prefrail state. Second, our study used two frailty models, which demonstrated that the accumulated disadvantages can endanger the elderly in the long term. Frailty management is more than treating specific clinical syndromes and physiological risk factors. Third, previous studies have identified that health behavior and management can be useful to delay or reverse frailty, such as physical activity, nutrition and rehabilitation [77]. The relationship between loneliness and frailty transition indicated that psychological treatment is also worthwhile for frailty intervention in older adults. Effective loneliness interventions can delay the progression of frailty. Finally, the findings on gender differences in our study suggest that attention should be given to older men with loneliness and its adverse outcomes.

The study has several limitations. First, among the total participants, 68% had completed data on the frail scale and 63–67% on the frail index in the two survey periods. Those who did not complete the questionnaire tended to be frailer and lonelier. Our findings may underestimate the relationship between frailty transition and loneliness. Second, as mentioned earlier, only one question concerned loneliness in CLHLS, likely underrating the prevalence of loneliness. However, a single question concerning loneliness has been widely used in studies and is more feasible for older adults to understand the investigation of loneliness [78, 79]. Finally, mental health variables, such as depression, were not included in the CLHLS.

## Conclusion

Our study examined the association between loneliness and frailty transition among older people in China and attempted to explore gender differences in the relationships. The results revealed that loneliness at baseline may lead to a reduced possibility of remaining in the robust or prefrail frailty state and that greater loneliness is associated with an increased risk of worsening frailty and remaining frail. The association between loneliness and frailty transition differs obviously between men and women. These findings should be considered when designing and implementing health and social care policies for older people.

## Data Availability

The datasets that support this article are publicly available from the project of the CLHLS. Questionnaires are free download at website (https://sites.duke.edu/centerforaging/programs/chinese-longitudinal-healthy-longevity-survey-clhls/survey-documentation/questionnaires/) and the datasets can be obtained after sending a data user agreement to the CLHLS team (https://sites.duke.edu/centerforaging/programs/chinese-longitudinal-healthy-longevity-survey-clhls/data-use-agreement/).

## Abbreviations

CLHLS: Chinese Longitudinal Healthy Longevity Survey
ADL: Activities of daily living
IADL: Instrument activities of daily living
